# The Carbon Catalogue, carbon footprints of 866 commercial products from 8 industry sectors and 5 continents

**DOI:** 10.1038/s41597-022-01178-9

**Published:** 2022-03-16

**Authors:** Christoph J. Meinrenken, Daniel Chen, Ricardo A. Esparza, Venkat Iyer, Sally P. Paridis, Aruna Prasad, Erika Whillas

**Affiliations:** 1 Columbia University, New York, NY USA; 2 CoClear, Purchase, NY USA; 3 University of Pennsylvania, Philadelphia, PA USA

**Keywords:** Climate sciences, Environmental impact

## Abstract

Product carbon footprints (PCFs) are playing an increasing role in decisions around sustainability for companies and consumers. Using data reported to CDP, we have previously built a dataset of 866 PCFs, from 145 companies, 30 industry groups, and 28 countries, showing trends of how upstream and downstream emissions vary by industry and how life cycle assessment (LCA) appears to aid companies in achieving steeper carbon reductions through improvements throughout a product’s value chain. Here, we present the greenhouse gas emissions and respective meta data for every product in this dataset. The Carbon Catalogue provides each product with name and description, PCF (in kg CO_2_e) and the respective LCA protocol/standard, product weight, as well as the name, industry, and country of incorporation of its manufacturer. For a subset of 421 products, the Carbon Catalogue further includes the PCF’s reported breakdown into two to nine separate stages of the product’s life cycle. For another subset of 250 products, the Carbon Catalogue includes how the respective PCFs changed and why the changes occurred.

## Abstract (summary)

**Table:** 

Measurement(s)	greenhouse gas
Technology Type(s)	Life Cycle Assessment
Factor Type(s)	LCA stage • Country • Company • Sector • Weight • Product Name
Sample Characteristic - Organism	Products (e.g., packaged consumer goods)
Sample Characteristic - Environment	Global Industrial System
Sample Characteristic - Location	Global

## Background & Summary

Most anthropogenic greenhouse gas emissions, henceforth GHG or simply “carbon”, are embedded in the life cycle of products we make and use – the cars we drive^1^, clothes we wear^2^, cloud-based data infrastructure we rely on^3^ buildings we live in^4,5^ and the food we eat^6,7^.

While the exact portion of total global GHG that is attributable to products has yet to be quantified directly, this portion has been estimated based on enterprise-level carbon accounting to be upwards of 75%^8^ or can be inferred from GHG by source: For example, 24% of global GHG arise from agriculture, forestry, or other land use, leaving 76% to industry, transport, and electricity & heat production^9^ – all of which can be traced to specific products such as the manufacturing and transportation of goods (e.g., a T-shirt) or their energy consumption once in use (e.g., a computer or furnace in someone’s home). In addition, a substantial portion of the 24% arise from cultivating crops and farm animals, which in turn is attributable to the GHG embodied in the resulting products, namely food^10^.

Based on this analysis by source, GHG that are embodied in products make up three quarters or more of all global GHG. Arguably, the increased awareness of the role of this product-embodied carbon^11^ gives rise to an emerging ecosystem of stakeholders, ranging from companies intending to include carbon labels on their products^12,13^, to consumer-oriented services, including financial institutions, aiming to inform consumers about their purchasing-related carbon footprint^14^, and finally consumers engaging in carbon-conscious purchasing^15,16^.

Each product’s embodied GHG, also referred to as its product carbon footprint (PCF) and commonly reported in units of mass of CO_2_e^17^, is a function of its entire life cycle: starting from its raw materials, its manufacturing process, then its transportation and use, and finally its waste/recycle management. This makes the life cycle of products, essentially their entire value chain, a crucial lever not only in assessing global GHG, but in reducing them^18^. As reviewed by O’Rourke (2014)^19^, the opportunities for the sustainable management of product value chains are vast, but many challenges remain. With respect to product carbon footprinting, noteworthy progress has been made, especially on three fronts: (i) More detailed “calculation rules”, often referred to as carbon footprinting standards or protocols, have removed ambiguities around how to determine the PCF of almost any product;^10,20–24^ (ii) qualitative and quantitative approaches to data quality have been formulated to better manage the uncertainty in PCFs^25,26^; and (iii) approaches borrowed from data science and machine learning have reduced the resources required of companies that carry out PCFs^7,26^. Further catalyzed by a momentum towards carbon labels, be it voluntary^13^ or through potentially forthcoming regulation^27^, the availability of PCFs has steadily improved and now includes a myriad of products and extensive underlying databases of raw materials and manufacturing processes (reviewed in Meinrenken *et al*.^18^).

Building on this momentum, in 2020 we built a dataset of 866 PCFs, from 145 companies, 30 industry groups in the *Global Industry Classification Standard* (GICS^28^), and 28 countries. This dataset shows trends of how the relative impact of upstream and downstream contributions to the PCF varies by industry, and how granular life cycle assessment (LCA) appears to aid companies in achieving steeper carbon reductions through optimizations throughout the product’s value chain^18^. The dataset was based on product carbon data that member companies of CDP (formerly the Carbon Disclosure Project) had reported – for public disclosure – to CDP in response to the Climate Change Questionnaire^29^, specifically to the LCA portion of the questionnaire’s supply chain module (henceforth “raw data”). Whether member companies quantified the PCFs primarily for the purpose of responding to CDP’s request, for internal management purposes, or for transparency to their customers, is not usually known. Informal conversations with some member companies suggest that PCFs are reported to CDP usually after they have already been assessed for other purposes. The dataset has since been used in other publications and white papers, such as the *World Economic Forum*’s report on the supply chain opportunity towards a net-zero carbon world^30^.

The database published herein^31^ makes this dataset available to everyone, at the level of each individual product. The database is rare for two reasons. First, rather than including the PCFs of generic, often not further identified products (e.g., “milk”, “LCD computer screen”), each product is identified with the actual company that made the product. Second, PCFs are presented in a consistent, uniform data structure which includes product weight, total PCF and its functional unit, breakdowns into life cycle stages, footprint changes, and various meta data. This allows for a wide range of analyses, including the carbon intensity (i.e., PCF per product weight)^18^; average PCF benchmarks by product type (e.g., cars, computer screens, etc.); trends in upstream vs. downstream emissions (by industry or over time); carbon hotspots^18^; how frequently companies typically update PCFs; and, perhaps most crucially, what strategic changes companies have implemented in order to reduce a product’s PCF. As such, the data published herein expands on our previous publication^18^ in two important ways: (i) The data is now available at the level of each individual product, rather than by, e.g., sector averages as published previously; and (ii) the data includes additional details and meta data, specifically: the CO_2_e at individual stages of the life cycle; the year of reporting; the country of the company that made the product, the product weight, its respective source, and the functional unit of the PCF; the protocol followed to quantify the PCF (e.g., GHGProtocol^10^); and finally the company-reported reason for any reported change in the PCF.

## Methods

As laid out in detail in Meinrenken *et al*.^18^, compiling the Carbon Catalogue broadly consisted of two main steps. Here, we expand our explanations of these steps to further aid users of the Carbon Catalogue, including of its more detailed raw and meta data that is published here but was not yet used in Meinrenken *et al*.^18^.

### Step 1

Organize and filter the product carbon data that member companies of CDP had reported for public disclosure to CDP (henceforth “raw data”). This step included mapping each company to one of eight broad industry sectors as well as mapping each reported life cycle stage to a uniform system of three value chain fractions, namely upstream, direct operations, and downstream.

### Step 2

Where not already supplied by the reporting company, identify the weight for each product.

This led to a series of 31 data fields for each product. Fifteen of these 31 fields show the raw data as submitted to CDP. The other fields represent our synthesis and inference of various portions of the raw data. These can be simple mathematical steps (e.g., the carbon intensity^18^ of a product), or systematic categorizations based on parsing of information that companies submitted in narrative form (e.g., the value chain fraction to which a reported life cycle stage belongs or the reason category for a reported change in PCF).

#### Data cleaning, identifying weights, and integrity screening

For the five years captured in the database (2013–2017), CDP members reported 1,597 PCFs for public disclosure. Of these 1,597 PCFs, 194 PCFs were blank, i.e., without GHG data or even a product name. Of the 1,597 PCFs, 263 PCFs were for services (e.g., a night spent in a hotel). PCFs for services were excluded from the Carbon Catalogue, because, while valid LCAs, they cannot be easily compared to the footprint of physical products^18^. Finally, 197 reported PCFs were incomplete, i.e., a product name may have been specified (e.g., “office printer”) but without sufficient detail about the type or origin of the product to determine its weight. Of the 943 remaining PCFs, 361 were reported along with their weight. For the other 582 footprints, we identified the (gross) weight via third party sources (estimated accuracy ± 5–10%)^18^. Of the 943 PCFs, the carbon intensity of 77 was outside a realistic range and thus their data deemed unreliable. These PCFs were subsequently excluded from the dataset as outliers. This meant that 866 PCFs remained that were deemed broadly reliable according to various criteria (see *Technical validation*). In some cases, adjustments were made to the raw data, based on context reported by the company in the raw data. As a common example, PCFs were meant to be reported in kg CO_2_e (as per guidelines of the CDP questionnaire^29^) but parsing the narrative information in reported meta data for a certain product revealed that the footprint was actually in, e.g., metric tons of CO_2_e. For transparency, such “typos” in the raw data were adjusted and any such adjustments to the raw data were recorded in the separate field “adjustments to raw data” in the database.

#### Assigning sectors

The 866 PCFs were from companies comprising 30 different *GICS* industry groups. In order to allow for analyses by industry – without however ending up with unsuitably small sample sizes – PCFs were mapped to a higher-level taxonomy of eight different industry sectors. The mapping is explained and available in Meinrenken *et al*.^18^ or can be gleaned directly from the database, which lists every PCF along with the original GICS identification and the assigned sector.

#### Breakdown to life cycle stages and mapping to three value chain fractions

For 454 of the 866 PCFs, companies reported, in addition to the total product’s carbon emissions, a breakdown of these emissions by different life cycle stages. As common in LCA, the number of separate stages varied, from two to nine per product. For 33 of these 454 PCFs, the sum of emissions reported at stage level were outside a 90–110% tolerance range^18^ vis-à-vis the total reported footprint. The stage-level data of these PCFs was therefore deemed unreliable and excluded from the database. In the raw data, companies used 312 different descriptions of these life cycle stages. In order to allow for meaningful analysis and comparison across products, these stage descriptions were mapped into one of three uniformly defined value chain fractions of the life cycle, each giving the respective GHG as a percentage of the total PCF: (i) upstream (i.e., GHG from raw material acquisition, pre-processing, and inbound transportation from suppliers); (ii) direct operations (i.e., GHG from the operations of the reporting company itself); and (iii) downstream (i.e., distribution to market, retail operations, use phase, and waste management). In addition, where possible, each of the 312 reported life cycle stages was identified as exclusively comprising (a) transportation; and/or (b) end-of-life (i.e., landfilling, recycling, or incineration of waste). This resulted in 421 of the 866 PCFs that provided enough information in the raw data to allow for a breakdown of the total GHG into at least two of said three value chain fractions. PCFs that emerged from this mapping with only upstream and direct operation emissions (but 0% downstream emissions) were for products which had been reported as cradle-to-gate footprints^10^. The value chain breakdown for PCFs that emerged from this mapping as having 0% upstream emissions was corrected such that the fraction originally mapped to direct operations was split into upstream and direct operation, according to the average respective split for all other PCFs in the same sector^18^. For transparency, these PCFs are indicated in the database by a separate field (%*upstream estimated from %operations – yes/no*). Of the 421 footprints, 25 were reported with one life cycle stage having negative CO_2_e, indicating offsets due to recycling^10^. We excluded these specific stages (i.e., one stage-level data point for each of the 25 PCFs) from the mapping to the three value chain fractions, for two reasons: First, they were typically small (up to ~5% of the total reported PCF, in other words below typical thresholds of materiality for PCFs^20,26^). Second, how to account for recycling offsets in a total PCF is still a subject of debate^32^ and governed by rigorous guidelines as to the quality and re-use of the recycled resource^10^. However, to retain full transparency of the reported raw data, the carbon emissions of all stages of said 25 products, including the stage with negative emissions, are included in the database, and the total PCF is left as reported by the company, regardless of any offsets the company may have included in the total PCF or not.

#### Reason categories for PCF changes

Since some PCFs were reported by the company along with a change in PCF (typically within the one to two years prior to reporting) and the reason for that change (provided by the company in narrative form), every PCF was assigned one of six change reason categories (four categories for the 250 PCFs that included a reported change and two categories for the other 616 PCFs):

1. PCF change reported, as due to actual GHG emission changes in the life cycle of the product (166 of 866 products)
2. PCF change reported, as due to model and/or parameter updates (25 of 866 PCFs)
3. PCF change reported, as due to a combination of (1) and (2) (21 of 866 PCFs)
4. PCF change reported, but reason for change not reported (38 of 866 PCFs)
5. No PCF change reported, with no provided reason (482 of 866 PCFs)
6. No PCF change reported, with clarification that no previous data was available (134 of 866 PCFs).

As shown previously, the above categorization of data can be used, for example, to infer to what extent LCA appears to aid companies in achieving steeper carbon reductions through improvements throughout a product’s value chain^18^.

## Data Records

### Data record glossary

The Carbon Catalogue database^31^, available on Figshare, is organized as a relational database in an easily accessible spreadsheet (*Microsoft Excel*). It consists of 25 product-level data fields in one data table (“Product Level Data”) and six life cycle stage-level data fields in another data table (“Stage Level Data”). All 31 fields are summarized as a glossary in Table 1, which, for convenience, is also included in the published database.

**Table 1:** Data record glossary for the Carbon Catalogue database, listing the 26 fields available at product-level (for “Main dataset” in Table 2) and five fields available at life cycle stage-level (for “Subset 1” in Table 2).

Fieldname	Table	Source	Type
PCF-ID	Product & stage level	Added during study	Text
Year of reporting	Product level	Raw data as reported to CDP	Numeric
Stage-level CO2e available	Product level	Added during study	Text
Product name (and functional unit)	Product level	Raw data as reported to CDP	Text
Product detail	Product level	Raw data as reported to CDP	Text
Company	Product level	Raw data as reported to CDP	Text
Country (where company is incorporated)	Product level	Raw data as reported to CDP	Text
Company’s GICS Industry Group	Product level	Raw data as reported to CDP	Text
Company’s GICS Industry	Product level	Raw data as reported to CDP	Text
Company’s sector	Product level	Added during study	Text
Product weight (kg)	Product level	Raw data as reported to CDP	Numeric
Source for product weight	Product level	Added during study	Text
Product’s carbon footprint (PCF, kg CO2e)	Product level	Raw data as reported to CDP	Numeric
Carbon intensity	Product level	Added during study	Numeric
Protocol used for PCF	Product level	Raw data as reported to CDP	Text
Relative change in PCF vs previous	Product level	Raw data as reported to CDP	Numeric
Company-reported reason for change	Product level	Raw data as reported to CDP	Text
Change reason category	Product level	Added during study	Text
%Upstream estimated from %Operations	Product level	Added during study	Text
Upstream CO2e (fraction of total PCF)	Product level	Added during study	Numeric
Operations CO2e (fraction of total PCF)	Product level	Added during study	Numeric
Downstream CO2e (fraction of total PCF)	Product level	Added during study	Numeric
Transport CO2e (fraction of total PCF)	Product level	Added during study	Numeric
EndOfLife CO2e (fraction of total PCF)	Product level	Added during study	Numeric
Adjustments to raw data (if any)	Product level	Added during study	Text
Description of LCA stage	Stage level	Raw data as reported to CDP	Text
Scope-characterization of LCA stage	Stage level	Raw data as reported to CDP	Text
Assigned value chain portion	Stage level	Added during study	Text
Emissions at stage (kg CO2e)	Stage level	Raw data as reported to CDP	Numeric
Emissions at this stage exclusively transport	Stage level	Added during study	Text
Emissions at this stage exclusively EndOfLife	Stage level	Added during study	Text

The field *PCF-ID*, a unique key for each of the 866 PCFs in the database, is used to map the product-level data (one row per PCF) to the stage-level data (2–9 rows per PCF). For convenience, the publicly available version of the database^31^ includes a copy of the glossary table, along with a 5^th^ column which includes detailed explanations of the range of possible values and meaning of each field.

For each PCF, we assigned a unique key within the database (*PCF-ID*) for two purposes: (i) to easily jump from the product-level data to the stage-level data; and (ii) to provide users with an indication of whether a particular company reported the PCFs of the same (or nearly same) product in multiple years. The latter is achieved by providing *PCF-ID* as a concatenation of three components: a company identifier, a product identifier, and the reporting year. Note that the product identifier was assigned solely based on parsing the reported product name (rather than a company-provided unique code which is not available in the raw data). This leads to rare cases where a product may have undergone a complete change from one year to the next, in essence creating a new product, but the product did not change its name and is thus captured as the “same” product in the database (same company and product identifier in *PCF-ID*). Similarly, it may lead to the opposite rare case where a company reports on the PCF of the same product over two years, but the reported name of the product changed, thus creating two products with separate product identifiers in the dataset.

In LCA, the impact is typically expressed per functional unit^33^. Functional units can be either single-use units, e.g., per one km driven in a car^1^, per one sheet of paper printed with a printer, per kWh of generated electricity^34^, or per feeding an infant for one day^6^. In other cases, functional units can be the entire life span of, e.g., a car, or the actual size of a purchased packaged food item, such as a 50 gram bag of potato chips^35^. In CDP’s LCA portion of the Climate Change Questionnaire^29^, companies were asked to specify the “Stock Keeping Unit” (rather than the functional unit) per which each PCF was reported (for example, “1 piece” for the product name “Keyboard”, “140 grams” for “Crisp’n light 7 grains” (see Fig. 3), or “1 kg” for “Sodium Bicarbonate”). In the Carbon Catalogue, the functional unit can thus be inferred from a combination of the two fields “product name” and “product weight”: For the majority of PCFs in Carbon Catalogue, the functional unit comprises the entire product over its life span (e.g., the printer with *PCF-ID* 10261-1-2017). In a minority of cases, notably for chemicals or construction items that are typically sold in bulk, the functional unit is a certain amount of a specific product (e.g., 1,000 kg of board for *PCF-ID* 16290-1-2013). In some cases, the field “product name” or “product description” contains additional text from the reporting company that further specifies the functional unit (e.g., “the functional unit has a life span of five years” for *PCF-ID* 1884-1-2013).

The stage-level data shows the raw, company-reported life cycle stages along with the respective CO_2_e for each stage (ranging from two to nine individual stages per PCF; average 4.2 stages per PCF). In addition to a general description of the life cycle stage (e.g., “Sugar beet supply - field preparation to factory gate”), the scope classification (1, 2, or 3) is included as well. While this scope classification originates in corporate carbon accounting^36^ and is not commonly used in LCA, a conceptual mapping between typical LCA stages and scope 1, 2, or 3 is possible^10^, and the LCA module of the CDP questionnaire^29^ includes this classification in order for a company to add further detail as to the nature of each reported life cycle stage (e.g., to differentiate scope 3-related “manufacturing” (i.e., by the reporting company’s suppliers) from scope 1&2 “manufacturing” (i.e., by the reporting company itself)). The raw data on life cycle stages is provided in the Carbon Catalogue database in order to allow for as detailed as possible analyses by the research community. However, in most cases the taxonomy of life cycle stages from one PCF to the next is not uniform, thus complicating comparisons across products and sectors. This is the reason why we mapped the information into the uniformly defined, three value chain fractions upstream, direct operations, and downstream, which each give the respective GHG as a percentage of the total PCF. These fractions are shown in the product-level data table.

### Overview of database and types of data granularity

As shown in Table 2, the 866 PCFs fall into five types, each characterized by the detail of information available for each PCF. All 866 PCFs contain the product’s total embodied carbon emissions and the product’s weight (in addition to the product’s name and description, as well as the name, *GICS*^28^, sector, and country of incorporation of the manufacturing company). A subset of 421 PCFs further includes information about the breakdown of the total carbon emissions by different stages of the life cycle. Of these 421 PCFs, 80 PCFs are based on a cradle-to-gate^10^ assessment (i.e., the product’s downstream emissions were not assessed and/or reported by the company). As expected, cradle-to-gate PCFs occur preferentially for chemicals, packaging for consumer goods, and, to a lesser extent, for construction and commercial materials^18^. Another subset of 250 of the 866 PCFs was reported along with a recent change in the product’s carbon emissions (typically one to two years prior to the report^18^). Finally, for 212 of these 250 PCFs, the company provided a detailed reason why the PCF changed. These reasons, in narrative form, are included in the database as well.

**Table 2:** Overview of the granularity types in the Carbon Catalogue database, arranged by detail of available information.

Detail included with footprint	Main:PCF	Subset 1: PCF & value chain breakdown			Subset 2: PCF & footprint change	
	Product weight and CO_2_ eq	Upstream, direct operations, downstream	Separate contribution for transport	Separate contribution for end-of-life	Footprint change	Footprint change and reason for change
Automobiles & components	75	12 [0]	4	10	7	5
Chemicals	116	39 [28]	14	0	42	30
Commercial equipm. & capital goods	56	35 [0]	31	20	19	19
Computer, IT & telecom	253	161 [8]	141	104	54	44
Construction & commercial materials	67	44 [17]	27	0	45	41
Food & beverage	139	70 [3]	67	14	54	50
Home durables, textiles & equipm.	122	35 [1]	12	32	23	17
Packaging for consumer goods	38	25 [23]	2	0	6	6
**All sectors (number of PCFs)**	**866**	**421 [80]**	**298**	**180**	**250**	**212**

Reproduced from Meinrenken *et al.*^18^ […] (in the third column from left) indicate the number of footprints that are cradle-to-gate^10^, i.e., footprints whose emissions downstream of the company’s own operations were not determined and/or reported. Of the 866 PCFs, 421 PCFs include a breakdown of the value chain into materials and processes related to upstream, direct operations, or downstream. Of the 421 PCFs, 298 PCFs further break out emissions specifically related to transport (which are also included in any of the three main value chain portions). Of the 421 PCFs, 180 PCFs further break out emissions specifically related to end-of-life (which are also included in the downstream portion).

### Example PCFs

In addition to the data glossary and the data at product-level and life cycle stage-level, the publicly available database includes a PCF viewer in order to provide users of the data with an easy mechanism to instantly display all numerical and narrative data available for a chosen PCF in one place. Below we use the output from this viewer to show three examples of PCFs, drawn from three of the above mentioned five PCF granularity types.

**Fig. 1:**
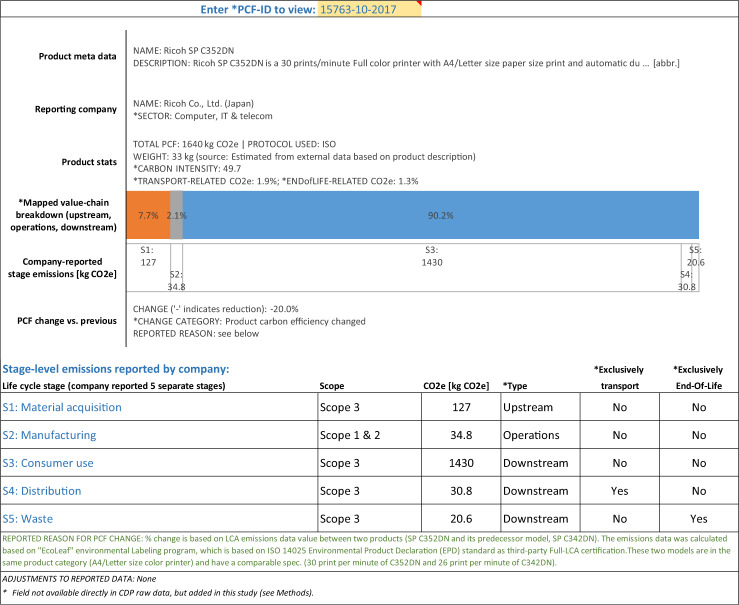
Example of cradle-to-grave PCF, reported with life cycle stage-level breakdowns as well as separately quantified transportation and end-of-life emissions.

Figure 1 shows an example of a PCF which was reported with stage-level data, which (in this particular case) included not only the usual upstream, direct operations, and downstream data but also further detail of the transport related emissions and end-of-life related emissions. Note that transport and end-of-life related emissions, even if separately identified and therefore quantified as such in the product-level data, are still counted towards the respective three value chain fractions. In other words, the three value chain fractions for every product add up to 100%, even if transport and/or end-of-life are separately quantified. The PCF in Fig. 1 was further reported to have undergone a 20% reduction in carbon emissions, due to actual changes in the product’s life cycle carbon emissions vis-à-vis its predecessor (as opposed to mere updates to the LCA methodology and/or parameters).

**Fig. 2:**
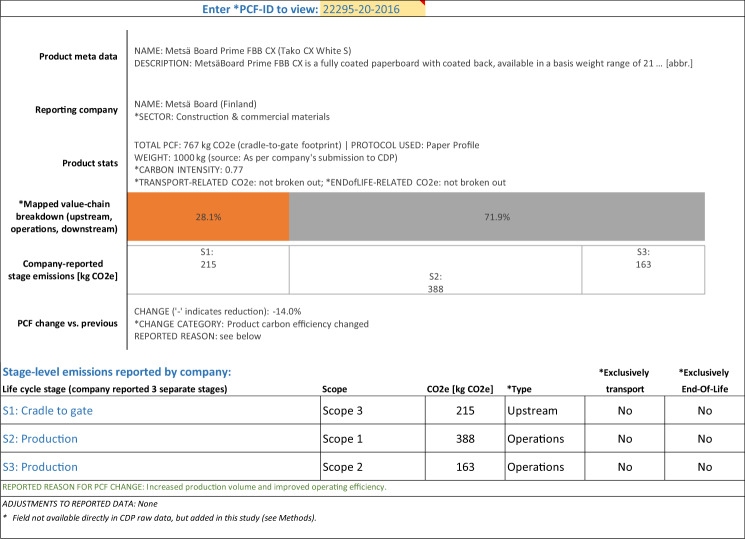
Example of cradle-to-gate PCF, reported with life cycle stage-level breakdowns.

Figure 2 shows an example of a PCF which was reported with stage-level data. However, the absence of reported downstream emissions indicates that this is a cradle-to-gate^10^ footprint. Emissions from (upstream) transportation are not separately identified (but included in total upstream emissions). This PCF was further reported to have undergone a 14% reduction in carbon emissions, due to actual changes in the product’s life cycle carbon emissions (in this case increased production volume and improved operating efficiency).

**Fig. 3:**
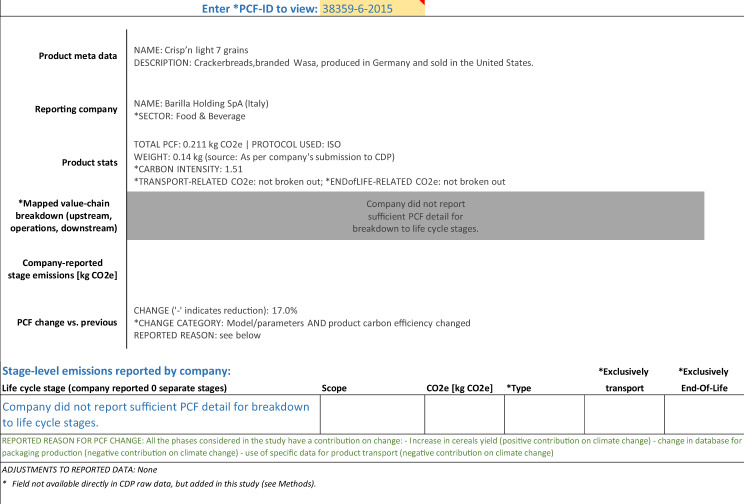
Example of PCF that was reported with insufficient or inconsistent stage-level data.

Finally, Fig. 3 shows an example of a PCF which was reported with insufficient or inconsistent stage-level data. This PCF was reported to have increased by 17%, due to a combination of actual changes in emissions (here: updated ingredients) and updates to the LCA methodology/parameters (here: updated LCA database for packaging materials).

### Characterization of industrial and geographic coverage in Carbon Catalogue

The database includes products from companies comprising a wide range of 30 *GICS*^28^ industry groups, including consumer apparel, cars, computers, food, and B2B products such as aluminum sheets. Table 3 shows an overview of the GICS classifications that are represented in the database, along with the mapped industry sector (see *Methods*) and the respective number of PCFs.

**Table 3:** Overview of the *GICS*^28^ classifications with PCF presentation in the database, along with the mapped industry sector (see *Methods*) and the respective number of PCFs in the database.

GICS Industry Group	GICS Industry	Number of products
Automobiles & Components	Auto Components	38
Automobiles & Components	Automobiles	13
Automobiles & Components	Unknown (field not used in 2013 data)	22
Capital Goods	Aerospace & Defense	1
Capital Goods	Building Products	6
Capital Goods	Electrical Equipment	14
Capital Goods	Machinery	5
Capital Goods	Trading Companies & Distributors	7
Chemicals	Unknown (field not used in 2013 data)	29
Commercial & Professional Services	Commercial Services & Supplies	42
Consumer Durables & Apparel	Household Durables	21
Consumer Durables & Apparel	Textiles, Apparel & Luxury Goods	31
Consumer Durables, Household and Personal Products	Unknown (field not used in 2013 data)	8
Containers & Packaging	Unknown (field not used in 2013 data)	8
Electrical Equipment and Machinery	Unknown (field not used in 2013 data)	11
Energy	Oil, Gas & Consumable Fuels	5
Food & Beverage Processing	Unknown (field not used in 2013 data)	13
Food & Staples Retailing	Food & Staples Retailing	14
Food & Staples Retailing	Unknown (field not used in 2013 data)	10
Food, Beverage & Tobacco	Beverages	70
Food, Beverage & Tobacco	Food Products	30
Food, Beverage & Tobacco	Tobacco	1
Forestry, Timber, Pulp and Paper, Rubber	Unknown (field not used in 2013 data)	13
Gas Utilities	Unknown (field not used in 2013 data)	1
Household & Personal Products	Personal Products	2
Materials	Chemicals	82
Materials	Construction Materials	1
Materials	Containers & Packaging	28
Materials	Metals & Mining	16
Materials	Paper & Forest Products	34
Media	Media	11
Media	Unknown (field not used in 2013 data)	4
Mining - Iron, Aluminum, Other Metals	Unknown (field not used in 2013 data)	3
Pharmaceuticals, Biotechnology & Life Sciences	Life Sciences Tools & Services	3
Retailing	Specialty Retail	2
Retailing	Unknown (field not used in 2013 data)	2
Semiconductors & Semiconductor Equipment	Semiconductors & Semiconductor Equipment	4
Semiconductors & Semiconductors Equipment	Unknown (field not used in 2013 data)	3
Software & Services	IT Services	1
Software & Services	Software	14
Software & Services	Unknown (field not used in 2013 data)	12
Technology Hardware & Equipment	Communications Equipment	7
Technology Hardware & Equipment	Computers & Peripherals	70
Technology Hardware & Equipment	Electronic Equipm., Instrum. & Components	47
Technology Hardware & Equipment	Office Electronics	24
Technology Hardware & Equipment	Unknown (field not used in 2013 data)	47
Telecommunication Services	Diversified Telecomm. Services	4
Telecommunication Services	Unknown (field not used in 2013 data)	4
Telecommunication Services	Wireless Telecommunication Services	1
Textiles, Apparel, Footwear and Luxury Goods	Unknown (field not used in 2013 data)	16
Tires	Unknown (field not used in 2013 data)	2
Tobacco	Unknown (field not used in 2013 data)	1
Trading Comp. & Distrib. and Comm. Serv. & Supplies	Unknown (field not used in 2013 data)	6
Utilities	Gas Utilities	2
	**Total products**	**866**

The countries of incorporation of the manufacturers of the products represented in the database comprise five continents (Table 4). More than half of the 866 PCFs are from manufacturers incorporated in three of the world’s five largest economies (USA, Japan, and Germany). However, a good representation of the other two top five economies is lacking, with only six PCFs for China-based companies and none for India.

**Table 4:** Overview of the countries of incorporation of the manufacturers of the products represented in the database, along with the respective number of PCFs.

Country	Products
USA	305
Japan	110
Germany	67
Taiwan	60
Netherlands	35
Finland	35
United Kingdom	32
Switzerland	28
Sweden	26
Italy	23
South Korea	22
France	20
Brazil	17
India	16
Spain	13
South Africa	11
Belgium	8
Canada	6
China	6
Australia	6
Ireland	6
Malaysia	4
Chile	3
Colombia	2
Luxembourg	2
Greece	1
Lithuania	1
Indonesia	1
**All 28 countries**	**866**

### Organization of the publicly available file

The Carbon Catalogue database^31^ is available as a standard spreadsheet file (*Microsoft Excel*). The main two tabs form a relational database of product-level data on one tab (one row for each of the 866 PCFs) and life cycle stage-level data on the other tab (two to nine rows per product; only for those 421 PCFs whose submissions to CDP included sufficient and internally consistent stage-level emission data; see *Methods*). The product-level and stage-level data are linked through a unique key, the *PCF-ID*. In addition, the spreadsheet includes a data glossary (see Table 1) as well as a data viewer which automatically generates, for any chosen PCF, a representation of all numerical and narrative data for a chosen PCF (see examples in Figs. 1, 2 and 3).

## Technical Validation

The scope for technical validation of the data was limited because each PCF was self-reported (to CDP) by the manufacturer of the respective product. Direct verification of a PCF or even parts of a PCF would require access to detailed underlying LCA inventory data^10^ (e.g., how much electricity was used in a specific manufacturer’s factory), which is not typically publicly available. In addition, biases in the data, e.g., a possible temptation by companies to report, for public disclosure, reductions in PCFs while choosing not to report in case a PCF increased, cannot be entirely ruled out and have been discussed along with our previous analysis of the data^18^. This principal limitation notwithstanding, below we summarize three aspects of the data which represent at least indirect approaches to verification and which give us confidence that the data in the Carbon Catalogue database^31^ can be considered broadly accurate and reliable. For a detailed discussion of possible reporting biases and representativeness of the products in Carbon Catalogue, please refer to Meinrenken *et al*.^18^ (section *Limitations and future* work).

### Data integrity screening

As summarized in *Methods* and explained in more detail in our previous analysis of the data^18^, we subjected the raw data that companies reported to CDP to a number of heuristic integrity screens, with respect to both the raw data’s agreement with available external benchmarks and its internal consistency. This led to the removal of 8% of reported PCFs because the reported carbon intensity was lower or higher than what could be realistically expected. Furthermore, the details of stage-level carbon emissions for 7% of products were removed because the sum of the reported stage-level emissions did not match the reported total PCF. Finally, we list in the database any adjustments to the raw data along with each PCF. A typical example of such an adjustment is that the CDP questionnaire^29^ asks for the CO_2_e figure to be submitted in kg, however a separate narrative submitted by the company makes it clear that the CO_2_e figure they submitted is in fact in metric tons. As detailed in Meinrenken *et al*.^18^, such adjustments were only made in cases where multiple aspects of the company-reported data provided near certainty of what the data was intended to convey. In contrast, in cases where doubt remained, we erred on the side of caution and removed the PCF from the database altogether.

### LCA protocols followed in determining the PCFs

As can be seen from Table 5, 70% of all reported PCFs followed one of the three major commonly recognized protocols, such as the ISO standard^23,24^, the GHG Protocol^10^, or PAS2050^20,21^. Another 9% followed one of the more bespoke standards (which are themselves broadly compliant with ISO). The 21% of PCFs for which the reporting company left the respective questionnaire field blank may be less reliable, because a reporting bias cannot be ruled out in all cases (i.e., the field was intentionally left blank because the PCF was determined without adhering to all pertinent rules).

**Table 5:** Overview of the carbon footprinting and/or wider LCA standards that companies reportedly followed in determining each PCF.

Standard/protocol	Products
ISO	40.8%
GHGP	21.1%
*NOT REPORTED*	*20.7%*
PAS2050	8.4%
TRACI 2.1	2.4%
Paper Profile	1.4%
MIT PAIA	1.0%
ILCD Handbook	0.9%
M. Env. Japan	0.5%
EcoLeaf	0.5%
EUPEF	0.2%
Carbon Labeling Certificate	0.2%
CEPI	0.2%
EICC Tool	0.2%
Japan CFP	0.2%
CFP Japan	0.1%
EPD	0.1%
French PEF	0.1%
Korea CL Guide	0.1%
M. Env. Japan v2.2	0.1%
Bilan Carbone	0.1%
WBCSD	0.1%
JEC/BIOGRACE	0.1%
JRM Assoc.	0.1%
FEFCO	0.1%
**All 866 products**	**100%**

70% of all reported PCFs followed one of the three major commonly recognized protocols. Another 9% followed one of the more bespoke standards (which are themselves broadly compliant with ISO^23^).

### Verification/assurance of the reported PCFs

A more nuanced picture emerges when considering the companies’ responses to CDP’s question whether the reported product emission data had been verified or assured (as encouraged by ISO^23^). The response rate to this question was low; only about one out of three PCFs included a response at all. This may be partially due to the fact that the question was asked at the level of life cycle stage emissions instead of for the PCF as a whole. Third party reviews of LCAs would usually be carried out either for all stages of the life cycle or for none at all^10^. This idiosyncrasy in the questionnaire could have led to possible confusion in this particular data item and therefore to companies simply leaving the response blank. Of the one in three PCFs that did include information about verification/assurance, 66% had been reviewed externally, 22% internally, and 3% had undergone a limited review. Only 9% had not been reviewed or assured at all, according to the reporting company. While this indicates fairly high robustness of the reported data, it must be considered likely that some companies chose to leave the question blank, precisely because the PCF had in fact not been verified/assured, thus creating a reporting bias in this particular data item. Because of the resulting uncertainty in this data item, the Carbon Catalogue database does not include the raw data on verification/assurance, instead only summarizing the findings here.

## Usage Notes

The Carbon Catalogue database^31^ is freely available for download by all interested users, as a simple *Microsoft Excel* file. For transparency, each data field indicates whether it represents the raw data that a company reported to CDP or the authors’ synthesis/inference of the raw data (see Table 1). The database allows for a wide range of analyses, including the carbon intensity (i.e., PCF per product weight)^18^, trends in upstream vs. downstream emissions (by industry or over time), carbon hotspots^18^, how frequently companies typically update PCFs and, perhaps most crucially, what strategic changes they implement in order to reduce a product’s PCF and how high the achieved carbon reductions were in each case.

The database is meant to be accessed directly via the two tabs “Product Level Data” and “Stage Level Data”, which are explained in section *Data Records*. In order for first time users to quickly familiarize themselves with the data structure, the *Microsoft Excel* file includes an additional tab that features a viewer where all data fields in the database can be viewed (but only for one product at a time). In addition, an interactive visualization of the database, however with far less detailed data on GICS^28^ industry sectors, life cycle stages, and transportation/end-of-life emissions, is available at CarbonCatalogue.coclear.co.

We would like to emphasize that, other than the systemization and inferences of the data described herein, the original calculations of PCFs were carried out by each reporting company itself. Therefore, for detailed questions about e.g., assumptions and boundaries in the PCFs that cannot be answered from the meta data of each product in the database, readers are referred to the respective reporting company.

## Data Availability

No custom code was used in assembling the dataset published herein. All steps of analysis and data processing are described in *Methods* as well as in Meinrenken *et al*.^18^.
